# Dairy Food at the First Occasion of Eating Is Important for Total Dairy Food Intake for Australian Children

**DOI:** 10.3390/nu6093878

**Published:** 2014-09-23

**Authors:** Malcolm D. Riley, Danielle L. Baird, Gilly A. Hendrie

**Affiliations:** Commonwealth Scientific and Industrial Research Organisation (CSIRO), P.O. Box 10041 Adelaide, South Australia 5000, Australia; E-Mails: Danielle.Baird@csiro.au (D.L.B.); Gilly.Hendrie@csiro.au (G.A.H.)

**Keywords:** children, dairy food, breakfast, Australia

## Abstract

The cross-sectional 2007 Australian National Children’s Nutrition and Physical Activity Survey collected detailed dietary information from a representative sample of more than 4400 children by 24-h dietary recall. Dairy food intake by Australian children is substantially lower than recommendations, and decreases as a percentage of energy intake as children grow older. Children aged 2 to 16 years are, on average, 2.3 times more likely to have a dairy food at the first daily occasion of eating, than at the second occasion. For children who consumed any dairy food at the first occasion of eating, the total daily intake of dairy foods was 129% (95% CI 120%–138%) greater than for children who did not consume a dairy food at the first occasion of eating. Their dairy food intake for the rest of the day following the first occasion of eating was also greater by 29% (95% CI 21%–37%). Younger age group, male sex, location of eating being at home or in a residence and starting the first occasion of eating from 6 a.m. to 9 a.m. are all jointly associated with having a dairy food at the first occasion of eating. A simple strategy to increase Australian children’s intake from the dairy and alternatives food group may be to make sure that the first occasion of eating each day includes a dairy food or a nutritional equivalent.

## 1. Introduction

Dairy foods contribute a significant percentage to a wide range of nutrients for children in different countries [1,2], and the importance of consuming dairy food in childhood and adolescence to maintain bone health has been established. Children who avoid drinking cow’s milk have reduced bone mineralization [3,4] and are at increased risk of bone fracture [5]. Women with a low milk intake during childhood have less bone mass during adult life and a greater risk of fracture [6].

The 2007 Australian national dietary survey of children indicates that the average amount of dairy food consumed by children aged 4 to 16 years is lower than the amounts recommended and decreases with age group [1]. An Australian longitudinal study [7] of children at the upper end of this age range (14 to 17 years) affirms a decrease in dairy food intake with age. Furthermore, there is evidence from Australia [8] and elsewhere [9] that dairy food intake by adolescents has decreased over recent years.

A large peak in children’s intake of dairy food occurs between 6 a.m. and 9 a.m. [1], indicating that breakfast time is an important occasion for children to consume dairy food. Breakfast is typically defined as the first meal of the day, eaten before or at the start of daily activities, within 2 h of waking and typically consumed no later than 10 a.m. [10]. However, there are many operational definitions of breakfast [11], often depending on naming of the eating occasion by subjects [12,13], or based on the time of consumption [9,14], or both [15]. Similarly “breakfast skipping” is often investigated where subjects are asked how often they skip breakfast without further definition of breakfast [16]. The importance of the definition of breakfast was demonstrated in an analysis where “breakfast skipping” was defined in 24 different ways with resultant variation in whether “breakfast skipping” was associated with indicators of overweight and obesity in Greek children [17]. The association was strongest when the definition of breakfast skipping was based on dietary behavior on the day of the survey, rather than reliance on reporting of past behavior or usual behavior.

Strategies are needed to help children meet their recommended intake for dairy foods, which have recently increased in Australia [18]. Breakfast is an important time for children’s dairy food consumption, but conceptualizations of breakfast are often subjective, dependent on meal composition, or arbitrarily restricted by time limits. Here we explored a data driven and objective definition of the “first daily occasion of eating”, which encompasses many other definitions of breakfast. This investigation sought to describe the first daily occasion of eating for Australian children, regardless of what time it occurs; determine its importance for dairy food intake and explore factors that are associated with consumption of dairy food at this occasion.

## 2. Experimental Section

### 2.1. Subjects and Dietary Data Collection

This analysis uses data collected as part of the 2007 Australian National Children’s Nutrition and Physical Activity Survey (ANCNPAS). The conduct of this survey was a collaborative effort between the Australian Commonwealth Scientific and Industrial Research Organisation (CSIRO) Preventative Health Flagship and the University of South Australia, with fieldwork managed by I-view Pty Ltd. (Sydney, NSW, Australia) The survey was jointly funded by the Australian Commonwealth Department of Health and Ageing, the Department of Agriculture, Fisheries and Forestry, and the Australian Food and Grocery Council. The survey objective was to assess food and nutrient intakes and physical activity patterns in a nationally representative sample of children aged 2–16 years.

The sample was randomly selected from across Australia using a quota-sampling scheme stratified by state or territory and by capital city/rest of state. Random clusters of postcodes were the primary sampling unit, from which eligible households were selected using random digit telephone dialing. The number of children selected from each state or territory was proportionate to the stratum specific population of children in that state or territory. The final response rate was 40%. Complete data sets were collected from 4487 participants or their caregivers from February to August 2007. Survey methodology and sample characteristics are described in detail elsewhere [19,20].

Trained interviewers collected dietary information using computer assisted, three-pass 24-h dietary recall methods, based on the dietary intake of the previous day (midnight to midnight). Two days of dietary intake were recalled, once by computer assisted personal interview and once by computer assisted telephone interview. Only the data collected by personal interview was used for these analyses. Dietary recall was provided by the primary caregiver for children 2–8 years and by the child for those aged 9 to 16 years. Where children were the primary source of information, the caregiver was encouraged to provide additional detail if required. The foods consumed were recorded with the time and location at which they were consumed. Information was not collected from the subject or the caregiver on what the eating occasion was named. A representative proportion of school and non-school days, and weekday and weekend days were collected across the sample.

Ethics approval was obtained for the survey from the Ethics Committees of the CSIRO (P21/06, September 2006) and the University of South Australia (P177/06, October 2006), which are registered with the Australian National Health and Medical Research Council. Permission to conduct secondary analyses was obtained from the Commonwealth Department of Health and Ageing.

For this analysis, an occasion of eating was defined to include all caloric food and drink consumed within an unlimited time interval provided there was not a gap of more than 60 min between any instance of caloric food or drink consumption. For each individual, all caloric food and drink consumed were assigned to an occasion of eating, which were ordered through the day as “first”, “second” and so forth. The following non-caloric foods were removed from the database prior to analysis: tea without milk (*n* = 332 instances of consumption), herbal tea (*n* = 56), coffee without milk (*n* = 29), soft drink or cordial with intense sweeteners (*n* = 337), mineral water (*n* = 29), carbonated or soda water (*n* = 42), tap, rain, tank or bore water (*n* = 8198), non sparkling bottled water (*n* = 2206).

AUSNUT 2007 is a food composition database specifically developed for the national survey, and containing 37 nutrients for 4225 foods, beverages and dietary supplements [21]. Dairy food was identified as any member of the major food group “Milk Products and Dishes” (MPD) which includes all dairy foods (e.g., plain and flavored milk, yoghurt, cheese, frozen milk products and custards) and mixed dishes where dairy foods are the major component (e.g., sweet sauces, mousse, fruche, creamed rice, and cheesecake). Butter is not included in MPD—it is categorized in the major food group “Fats and Oils”.

Intake of dairy food was expressed as serves to compare intake with government dietary recommendations [18]. The estimation of serves has been previously described [1] and is based on Australian government definitions [18] of dairy food reference serve sizes to be one cup (250 mL) of milk (fresh, long life or reconstituted dried milk), half a cup (125 mL) of evaporated milk, 40 g of hard cheese, and 200 g of yoghurt. The recommended average daily number of serves for dairy foods is 1.5 serves a day for 2–3 year olds, 1.5 serves and 2 serves for 4–8 year old girls and boys respectively, 3 serves and 2.5 serves for 9–11 year old girls and boys, respectively, 3.5 serves for 12–13 year olds and 3.5 serves for 14–16 year olds [18]. Only dairy food based on cow’s milk was included in these analyses—dairy substitutes (e.g., soy based foods) were consumed by 4% or less within any age/sex group above the age of four years [1].

### 2.2. Statistical Analysis

Statistical analyses were performed using STATA SE12 (StataCorp LP, College Station, TX, USA).

Population weights to account for the non-proportional sampling scheme of this survey were derived based on the age, gender and region distribution in the 2006 Census of Population and Housing, rescaled to the size of the sample for inferential statistics and were applied for all analyses.

Sex, day type, and location of eating were dichotomous variables with day type categorized as a school day, or a non-school day (weekend day or holiday) and location of eating categorized as at a residence or at another location (place of purchase, while travelling, at school). Time of beginning consumption was categorized into three groups (before 6 a.m., 6 a.m. to 9 a.m. and after 9 a.m.). Age group was categorized into four groups (2–3 years, 4–8 years, 9–13 years, and 14–16 years).

Chi squared tests were used to assess the evidence for a difference in sex, day type, time of first occasion of eating and place of first occasion of eating across age groups. If there was evidence for a difference between age groups, *post hoc* analysis for difference between groups was undertaken with Bonferroni adjustment. One-way analysis of variance was used to assess the evidence for a difference in total number of eating occasions on the day of survey across age groups followed by *post hoc* analysis between groups using Bonferroni adjustment. Logistic regression was used to assess differences between groups in the proportion of children consuming dairy foods at the first occasion of eating.

Linear regression models were used to estimate the difference in dairy food consumption (total daily, and on all occasions excluding the first occasion of eating) for those who consumed a dairy food on the first occasion of eating compared to those who did not consume a dairy food on the first occasion of eating. The dependent variable in these models were amount of dairy foods in serves summed across the relevant occasions, and the independent variables were sex, age group (2–3 years, 4–8 years, 9–13 years and 14–16 years) and a dichotomous variable identifying whether a child consumed a dairy food on the first occasion of eating or not.

Multiple logistic regression modeling was used to explore factors associated with eating a dairy food at the first occasion. Sex, day type, location of eating, age group and time of consumption were included as independent variables. Age groups were included as dummy variables with 2–3 years as the reference age group. Times of beginning consumption groups were also included as dummy variables with 6 a.m. to 9 a.m. being the reference time group. It was considered that socioeconomic factors might be related to whether a dairy food was consumed at the first occasion of eating in addition to the above factors. While the achieved educational level, and the current employment of the parents of the subjects was collected in the survey, for simplicity self-reported household income in tertiles was considered as an independent variable. Household income was available for 4251 subjects (95% of the sample). Results were considered statistically significant if *p* < 0.05.

## 3. Results

For most children in each age group, the beginning of the first daily occasion of eating was from 6 a.m. to 9 a.m., but this decreased in the older age groups (*p* < 0.001, Table 1). School days and non-school days were well represented for each age group, but for younger children the day on which the dietary recall took place was more likely to be a school day (*p* < 0.001, Table 1). Only 5.1% of children had their first daily occasion of eating at a place other than their home or another residence, and this became more common for older children (*p* < 0.001) (Table 1). There was no significant difference between boys and girls for any of the factors shown in Table 1 except for place where first occasion of eating occurred. A higher percentage of older girls than older boys were not located in a residence on their first occasion of eating, however both boys and girls showed a significantly higher percentage of first occasions of eating outside a home in older age groups.

Dairy food was reported to be avoided by 76 children (1.7%). No reason was stated for a dairy free or low dairy diet for 35 children (0.8%), while others stated reasons relating to dairy allergy or intolerance, or lactose intolerance (*n* = 41, 0.9%). The proportion of children who had dairy food on their first occasion of eating did not differ according to whether dairy food was stated to be avoided or not (*z* = 1.24, *p* = 0.21), and these children were therefore retained in the analysis.

**Table 1:** Characteristics of subjects and their first occasion of eating.

Characteristic	Age Group (Years)				
	2–3	4–8	9–13	14–16	*p* Value
*n*	1071	1216	1110	1090	
Sex (% girls)	49.7%	48.7%	48.9%	48.8%	*p* = 0.17
First occasion of eating before 6 a.m.	28 (2.6%)	12 (0.9%)	15 (1.3%)	32 (2.8%)	
First occasion of eating 6 a.m.–9 a.m.	993 (92.9%)	1131 (92.4%)	908 (83.2%)	735 (67.7%)	
First occasion of eating after 9 a.m.	50 (4.5%) ^a^	73 (6.7%) ^a^	187 (15.5%) ^b^	323 (29.5%) ^c^	*p* < 0.001
Day type (school day *versus* other *)	767 (73.0%) ^a^	709 (58.9%) ^b^	564 (51.1%) ^c^	551 (52.4%) ^c^	*p* < 0.001
First occasion of eating at home or other residence ^†^	1049 ^a^ (98.3%)	1177 ^a^ (96.6%)	1029 ^b^ (93.5%)	985 ^b^ (90.6%)	*p* < 0.001
Total eating occasions on the recall day	5 (1–8) ^a‡^	5 (2–9) ^b^	5 (2–8) ^b^	5 (2–8) ^c^	*p* < 0.001

Different superscript letters in rows indicate statistically significant differences on *post hoc* testing * “Other” includes weekend, public holidays and school holidays; ^†^ data was missing for the place of first occasion of eating for 3 children aged 2–3 years, 2 children aged 4–8 years, 8 children aged 9–13 years and 7 children aged 14–16 years; ^‡^ median (minimum, maximum).

**Figure 1:**
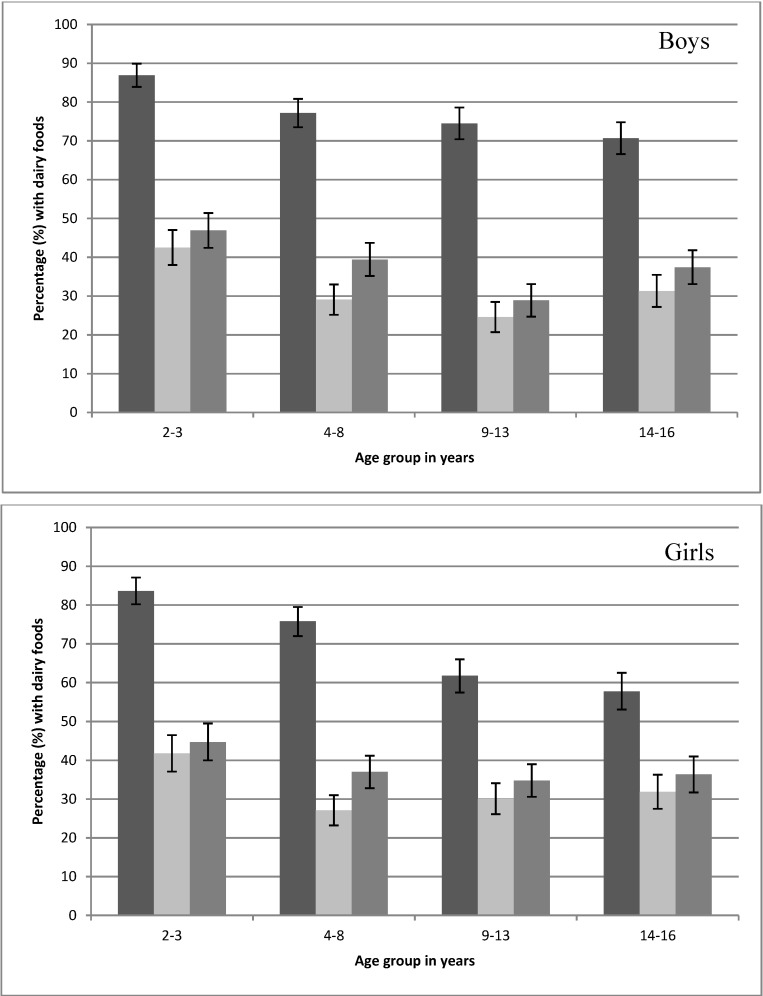
Percentage (±95% CI) of the first three eating occasions (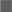 first eating occasion, 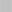 second eating occasion, 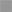 third eating occasion) that included one or more dairy foods on the day surveyed from the 2007 Australian National Children’s Nutrition and Physical Activity Survey (Computer Assisted Personal Interview only).

Examination of the first three eating occasions of the day by age group found that inclusion of a dairy food was highest at the first eating occasion (54%–82%) compared to 22%–40% for the second and third eating occasions (Figure 1). The median time of commencing each occasion of eating was later in the day for each age group, with the median time of commencing the third occasion of eating (*n* = 4461) being 12:30 p.m., 1:00 p.m., 1:15 p.m. and 2:00 p.m. for the age groups 2–3 years, 4–8 years, 9–13 years and 14–16 years, respectively.

The mean amount of dairy food consumed (in serves) by consumers was greater at the first occasion of eating than the second or third occasion for younger children (Table 2). For children aged 9 to 13 years or 14 to 16 years, there was generally no significant difference in mean consumption of dairy food between the first, second, or third occasion of eating.

**Table 2:** Overall mean dairy food intake and mean dairy food intake at each of the first three occasions of eating in consumers grouped by sex and age.

Occasion	Age Group (Years)			
	2–3	4–8	9–13	14–16
*Girls*				
Overall dairy food intake	2.17 ^†^ (2.06–2.29) 0.6%	1.89 (1.80–1.99) 5.5%	1.94 (1.83–2.05) 8.0%	2.01 (1.88–2.14) 9.3%
1st occasion	0.89 (0.84–0.95) 16.3%	0.87 (0.82–0.92) 24.4%	0.92 (0.86–0.98) 39.8%	0.97 (0.89–1.05) 41.8%
2nd occasion	0.69 (0.63–0.76) 58.9%	0.69 (0.61–0.77) 72.5%	0.77 (0.69–0.86) 68.9%	0.97 (0.84–1.10) 67.1%
3rd occasion	0.73 (0.66–0.81) 54.4%	0.73 (0.66–0.80) 62.4%	0.84 (0.75–0.94) 65.9%	0.92 (0.80–1.04) 64.3%
*Boys*				
Overall dairy intake	2.30 (2.19–2.40) 1.1%	2.11 (2.01–2.21) 4.7%	2.48 (2.34–2.62) 6.7%	2.81 (2.64–2.97) 5.0%
1st occasion	0.90 (0.85–0.94) 14.0%	0.94 (0.89–0.99) 22.3%	1.20 (1.13–1.27) 25.1%	1.37 (1.28–1.46) 29.2%
2nd occasion	0.75 (0.68–0.81) 58.3%	0.81 (0.72–0.90) 71.5%	1.14 (0.97–1.32) 73.7%	1.07 (0.95–1.19) 67.7%
3rd occasion	0.71 (0.65–0.77) 52.1%	0.78 (0.70–0.86) 60.6%	1.03 (0.91–1.16) 69.5%	1.12 (1.02–1.23) 60.8%

^†^ Mean dairy food intake in serves, (95% CI), % who did not consume dairy food. A serve of dairy food is 250 mL of milk, 200 mL of yogurt or 40 g of cheese.

**Figure 2:**
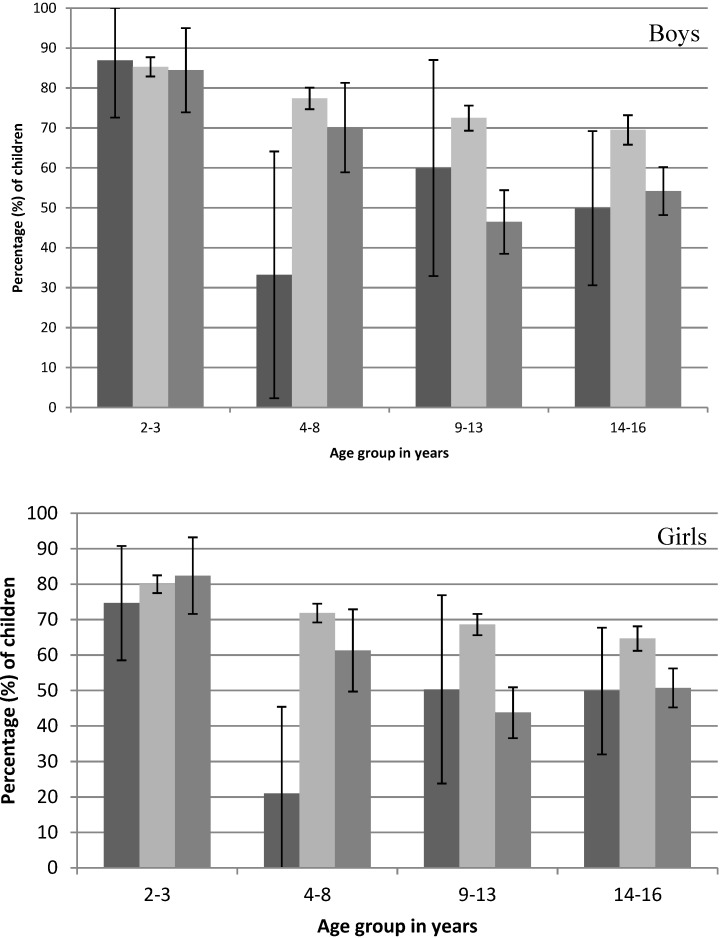
Percentage of children (±95% CI) where the first occasion of eating includes a dairy food by the start time of consumption (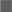 before 6 a.m., 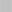 6 a.m. to 9 a.m., 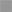 after 9 a.m.) on the day surveyed from the 2007 Australian National Children’s Nutrition and Physical Activity Survey (Computer Assisted Personal Interview only).

Young children (2–3 years) were most likely to include dairy at the first occasion of eating, regardless of what time this began. However, for children aged 9–16 years, when the first occasion of eating began after 9 a.m., the percentage of children who included a dairy food was significantly lower than when the first occasion of eating occurred from 6 a.m. to 9 a.m. (Figure 2). This pattern was consistent for boys and girls.

The percentage of children who had a dairy food on the first occasion of eating was 74.2% when the occasion occurred in a residence and 42.9% when it occurred at a place other than a residence (*p* < 0.001). On a school day, the percentage of children who had a dairy food on the first occasion of eating was 73.6%, non-significantly higher than the 70.7% who had a dairy food on the first occasion of eating on a non-school day (*p* = 0.07).

**Figure 3:**
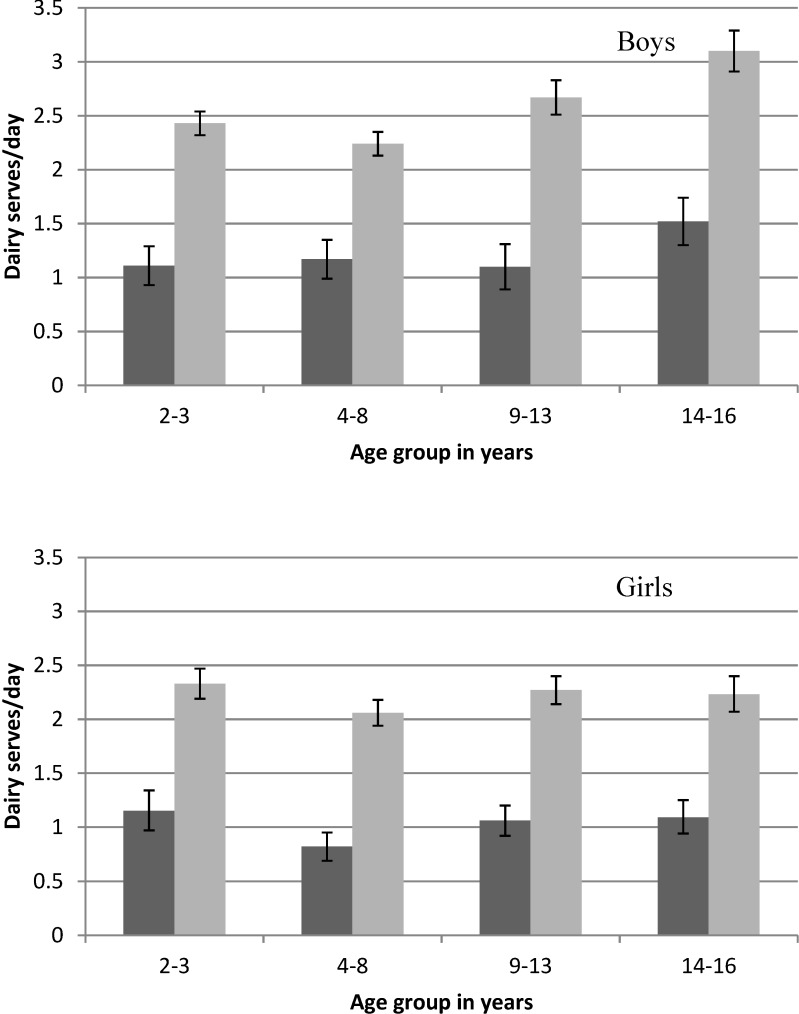
Mean daily serves of dairy food by sex, age group and whether dairy food was consumed at the first occasion (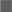 full day intake for children where no dairy food was consumed at the first eating occasion, 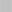 full day intake for children where dairy food was consumed at the first eating occasion) on the day surveyed from the 2007 Australian National Children’s Nutrition and Physical Activity Survey (Computer Assisted Personal Interview only).

Total daily serves of dairy food is higher for children who consumed dairy food at their first eating occasion, with the average amount consumed for all age groups being 129% (95% CI 120%–138%) higher than those who did not consume dairy at the first eating occasion (Figure 3). Nevertheless, the average dairy food intake for the 14–16 year old group was well below the 3.5 daily serves recommended. For 9–13 year olds, the dairy food intake recommendation varies from 2.5 serves a day for 9–11 year old boys to 3.5 serves a day for 12–13 year olds. The mean dairy food intake for 9–13 year olds was below the recommendation for all groups except 9–13 year old boys who consumed a dairy food at the first occasion of eating—the average intake of this group fell between the recommendation for 9–11 year old boys and 12–13 year old boys (Figure 3).

Children who consumed a dairy food at their first occasion of eating also consumed more dairy food for the remainder of the day compared to children who did not consume a dairy food on their first occasion of eating. Discounting the amount of dairy food consumed at the first occasion of eating, children who consumed a dairy food at their first occasion of eating also consumed an average of 29% (95% CI 21%–37%) more dairy food for the rest of the day than children who did not consume a dairy food on their first occasion of eating.

A serve of dairy food is 250 mL of milk, 200 mL of yogurt or 40 g of cheese.

A logistic regression analysis was conducted to determine the independence of factors associated with having a dairy food at the first occasion of eating (Table 3). The results indicate that younger age group, male sex, location of eating being at home or in a residence and starting the first occasion of eating from 6 a.m. to 9 a.m. are all jointly associated with being more likely to have a dairy food at the first occasion of eating. After controlling for the other factors, whether the day was a school day or a weekend or public holiday was not associated with whether a dairy food was consumed at the first occasion of eating.

**Table 3:** Multiple logistic regression for a dairy food being consumed by children (*n* = 4467) at the first eating occasion.

Variable	Coefficient (95% CI)	Standard Error	*Z*	*p* Value
Intercept	1.39 (0.94 to 1.84)	0.23	6.0	<0.001
Sex	−0.35 (−0.51 to −0.19)	0.08	−4.4	<0.001
Age 4–8 years	−0.57 (−0.81 to −0.33)	0.12	−4.6	<0.001
Age 9–13 years	−0.90 (−1.13 to −0.66)	0.12	−7.4	<0.001
Age 14–16 years	−0.94 (−1.19 to −0.70)	0.12	−7.7	<0.001
Before 6 a.m.	−0.75 (−1.28 to −0.22)	0.27	−2.8	0.006
After 9 a.m.	−0.59 (−0.81 to −0.37)	0.11	−5.3	<0.001
Day type	−0.007 (−0.15 to 0.17)	0.08	0.09	0.93
Location	0.99 (0.69 to 1.28)	0.15	6.5	<0.001

Reference category for age group is 2–3 years; and for time at beginning of consumption is from 6 a.m. to 9 a.m.; day type is coded 1 for non-school day, 0 for a school day; sex is coded 1 for girls, 0 for boys; location is coded 0 for consumption outside of home, 1 for consumption in own home or another residence.

For 4251 subjects, self-reported household income data was available. A positive association was found between household income grouped into three categories (less than $52,000; $52,000 to $103,999; and above $104,000) and consumption of dairy food at the first occasion of eating (Chi Squared = 13.44, *p* = 0.001). When this factor was included in the logistic regression model, all factors previously found to be significantly associated with consuming dairy food on the first occasion of eating remained significantly associated, with the low household income compared to high household income also being statistically significantly associated.

## 4. Discussion

The definition of eating occasion used here is novel, and has advantages over other definitions used. It is not dependent on consumption within a specific time interval, or on the individual judgment of each subject. The definition focuses on the boundary between eating caloric food or drink, and while the critical interval is arbitrarily set at one hour, the resulting distribution of total eating occasions in the day (Table 1) has face validity.

Regardless of what time it is consumed, the first occasion of eating is more likely to include a dairy food than subsequent occasions of eating for all age groups. For children 9–13 years and 14–16 years, the percentage who consumed a dairy food at the first occasion of eating was smaller if that occasion started after 9 a.m. but was still larger than the percentage who consumed a dairy food at the second or third occasion of eating in the day (44% and 51% compared to 24%–26% and 25%–29% for 9–13 years and 14–16 years, respectively). For children who consumed a dairy food at the first occasion of eating, not only was their total dairy intake much higher than children who didn’t consume a dairy food on their first occasion of eating, but ignoring the contribution of the dairy food consumed at breakfast, their dairy intake for the rest of the day was also substantially much higher. Consuming dairy food on the first occasion of eating is relatively widespread and its encouragement provides an excellent opportunity for addressing the current shortfall in dairy food intake in Australian children.

A greater prevalence of children in older age groups had their first occasion of eating after 9 a.m. This may be because extended morning sleep takes a higher priority than having breakfast in the older age groups. Total sleep duration decreases with age through childhood and adolescence [22], and this is probably a result of later bedtimes with age especially for adolescents [23]. The majority of adolescents get less than optimal sleep [24], and an association of inadequate sleep with energy-rich foods has been seen in Finnish 10–11 year old children [25]. In 9–16 year old Australian children, those who went to bed late also consume more energy-rich foods [26]. A plausible reason for these findings is that sleep deprivation may result in children sleeping longer in the morning and being less likely to eat at home prior to leaving for school. Different foods may be available for consumption at home compared to school, or during travel to school. An area for future research is the extent to which other foods consumed differ according to what time the first occasion of eating occurs—we have shown that dairy food is more likely to be consumed when the first occasion occurs earlier. For a sample of South Australian children aged 13–18 years, 77% were woken by an alarm or by their parents on school days compared to 9% on the weekend [23]. Appropriate parent-set bedtimes might be a practical step to increase sleeping time, which in turn may increase the prevalence of eating breakfast at home. Although only occurring for 17.5% of Australian adolescents, parent-set bedtime was one explanatory factor for a longer sleep time for adolescents in Australia compared to the US [27].

Multiple logistic regression showed that not having a dairy food at the first occasion of eating was jointly associated with being female, being in an older age group, starting the first occasion of eating before 6 a.m. or after 9 a.m. and eating in a different location than a residence. Therefore, while older children are more likely to start their first occasion of eating for the day outside the hours of 6 a.m. to 9 a.m. than younger children, this was not the only reason that a smaller percentage of older children have a dairy food on their first occasion of eating. The independent associations suggest that there are reasons other than propensity to start eating later in the day, and to eat outside of home on the first occasion of the day that also contribute to a low prevalence of dairy food intake on the first occasion of eating for older children. Household income could act as a surrogate for other factors related to socioeconomic factors. The fact that it is an independent determinant of consuming a dairy food at the first occasion of eating in multivariate analysis but did not substantially change the association observed for other factors indicates the robust nature of the associations with sex, age group, time of beginning consumption, and place of consumption.

Children who did not consume dairy food at the first occasion of eating consumed less dairy food throughout the rest of the day than children who consumed dairy food at the first occasion of eating. Children who miss having dairy food at their first occasion of eating do not “catch up” to meet their dairy recommendation by consuming dairy foods at other times of the day. Instead, children who do not have a dairy food at the first occasion of eating have a mean intake of dairy foods for each age group that is less than half of the intake of children who consumed a dairy food on their first occasion of eating. Similarly, among 4th to 6th grade students in the US, those who started the day with juice or fruit were more likely to consume a greater amount of fruit and vegetables [28]. Consumption of breakfast in the United States is strongly associated with total daily dairy food intake [29]. In Brazil, among 16–20 year olds, children who do not eat breakfast (definition not given) also consumed less than half the daily amount of dairy foods compared to children who do eat breakfast (112 g *vs.* 262 g, *p* < 0.001) [30].

It is unsurprising that children who do not have a dairy food at the first occasion of eating, do not match the dairy food intake of those who do. Reasons for not having a dairy food on the first occasion of eating may also apply throughout the day and might include having a known allergy to dairy foods, particular health beliefs and individual food preference. True allergy to cow’s milk protein has been estimated to be about 2% in infants and frequently resolves by two years of age [31]. Lactose maldigestion, due to loss of functioning lactase between the ages of 3–5 years, occurs in most humans [32] however lactose in usual intake of dairy foods are not a major cause of symptoms [33].

Other reasons for not choosing dairy at the first occasion may include lack of availability, their personal or other family member’s habits, which are not isolated to the first occasion of eating.

Health beliefs of parents may positively or negatively impact on dairy food intake. A positive belief about the health benefit of dairy foods is associated with an increased intake of dairy food amongst their children with parental attitude and influence being associated with dairy food intake in a range of cultures [34,35]. A negative parental belief about the benefit of dairy foods would be expected to have the opposite effect [36,37]. Personal health beliefs may have an increasing influence on food intake as children grow older, and may differ substantially between girls and boys. For example, gender differences in body image concerns appear between 8 and 10 years of age and become more pronounced with age particularly for girls [38]. Dairy food avoidance is associated with dieting frequency, particularly for adolescent girls [39]. In older women in New Zealand, an increase in body weight was given as an important reason for not increasing dairy food intake [40].

The importance of children’s preferences in determining their food intake has been widely accepted. In reviewing the area, Birch *et al.* [41] have highlighted the importance of early life exposure to different foods in determining food preference. Dairy foods (milk, cheese, yogurts, and custard) are very commonly consumed in early life but the intake of milk in particular relative to total dietary intake decreases with age. It seems likely that foods introduced into the diet and for which the child develops a preference, eventually displace foods, which formerly provided a larger percentage of dietary energy. It is possible that more preferred foods are partially displacing dairy foods for the first occasion of eating for the day. There is evidence from the United States [42] that dietary preference is established prior to high school and maintains its relative position within a peer group despite group change in preference (*i.e.*, dietary preferences “track” with time).

The availability of dairy food when it is required is potentially an important consideration. Expense of dairy foods and storage problems have been described as barriers to dairy food consumption by US college students [43], while accessibility is described as a facilitator. In the Australian context, when the first occasion of eating took place away from home, the eating occasion was less likely to include a dairy food. This might be due to lack of availability at the place of purchase or competition from other food, or lack of appropriate storage or inconvenience for foods carried from home. In Poland [44], it is estimated that 90% of 14 to 15 year old children eat “first breakfast”—the first meal of the day eaten at home and 50% of these include a dairy food. Second breakfast is consumed at school by 85% of the children but only 3% of these include a dairy food. Therefore, the availability of dairy foods in both the home and at school may be important enablers of dairy food consumption in children.

The habitual incorporation of dairy food into the first occasion of eating appears culturally appropriate in Australia because it is a dominant characteristic of this eating occasion regardless of the time at which it occurs. Breakfast consumption among young US adolescents (mean age 14 years) was significantly associated with whether their friends consumed breakfast [45]. Positive peer attitudes are also associated with having breakfast among 14-year-olds in Delhi, India [35]. Factors associated with having breakfast may apply more generally to the type of food consumed at the first occasion of eating. For US college students [43], focus groups have established that seeing others drink milk is a facilitator for dairy food intake.

The strength of these findings is that they arise from data collected in a large national sample of children using a standardized methodology. However, the data arises from a single day of recalled dietary intake, which may include misreporting of food intake in relation to factors we have examined such as age [46].

The average dairy food consumption for Australian children in the 2007 National survey is less than the recommended amount for all age groups from 4 to 16 years [1]. Having a dairy food at the first occasion of eating contributes about 50% (48%–52%) of the total energy from dairy food for the day to children in that age range. A recent leading article makes the point for US children that “those who avoid milk must plan a strategy for replacing the nutrients contributed by milk or they are likely to fall short of the recommendations for calcium and other nutrients” [47]. A similar point can be made for Australian children who do not consume a dairy food or its nutritional equivalent at their first occasion of eating—they are unlikely to meet the recommendations for dairy food intake. In fact, for the data we examined, the average daily amount of dairy food consumed by children who included a dairy food at the first occasion of eating was less than the current dairy food recommendation for children aged 12 years and older. Therefore, even though the inclusion of a serve of dairy food at the first occasion of eating is a good strategy to increase dairy food intake, additional strategies are also required to reach the recommended intake. Other factors also influence the consumption of dairy food and low consumption of dairy foods in adolescents has also been associated with low socioeconomic class, low family connectedness and low achievement at school [39], as has low intake of fruit and vegetables [48]. This suggests that patterns of unhealthful eating may be determined by similar underlying factors and be best addressed through integrated programs.

Interventions to increase daily dairy food intake have been successful in US college students over 8 weeks by a mean amount of 0.17 serves [43]. For younger children, a review of interventions [49] suggest that an increase in dairy food intake of one serve a day is achievable by focusing on dairy food only (rather than multiple foods or general dietary advice), improving the availability of dairy food, and by engaging the parents directly in the intervention.

While these results raise questions that might be usefully addressed by intervention studies, further descriptive research would provide insight. It may be useful to compare the patterns seen here for dairy food with other food groups [50], and to extend the exploration to occasions of eating throughout the day. The mechanism for the association of dairy food at the first occasion of eating with household income and with eating at home should be examined in much greater detail. The dietary patterns for the first occasion of eating may also show similar patterns for adults as those shown here, and the extent to which shared family environment is influential may be a fruitful area of work.

## 5. Conclusions

The first occasion of eating of the day is an important occasion for intake of dairy foods by children. Children who consumed a dairy food at the first occasion of eating, compared to children who did not, had a higher intake of dairy food for the rest of the day and a much higher overall daily intake of dairy foods. It has been recommended that a good quality breakfast should include a serve of fruit, cereal and dairy food [11,17]. A simple strategy that should be trialed to increase intake from the dairy and alternatives food group in Australian children is to make sure that the first occasion of eating each day includes a dairy food or its nutritional equivalent. This strategy should be focused especially on girls, for children when their first occasion of eating occurs after 9 a.m., and for children eating away from home.
